# Engineered ACE2 decoy in dry powder form for inhalation: A novel therapy for SARS-CoV-2 variants

**DOI:** 10.1016/j.omtm.2025.101459

**Published:** 2025-03-31

**Authors:** Takaaki Ito, Tatsuya Suzuki, Yusuke Sakai, Keisuke Nishioka, Yumi Itoh, Kentarou Sakamoto, Nariko Ikemura, Satoaki Matoba, Yasunari Kanda, Junichi Takagi, Toru Okamoto, Kohei Tahara, Atsushi Hoshino

**Affiliations:** 1 Laboratory of Pharmaceutical Engineering, Gifu Pharmaceutical University, Gifu 501-1196, Japan; 2 Department of Microbiology, Juntendo University School of Medicine, Tokyo 113-8421, Japan; 3 Research Institute for Microbial Diseases, Osaka University, Osaka 565-0871, Japan; 4 Department of Pathology, National Institute of Infectious Diseases, Tokyo 208-0011, Japan; 5 Department of Infectious Diseases, Graduate School of Medical Science, Kyoto Prefectural University of Medicine, Kyoto 602-8566, Japan; 6 Laboratory for Protein Synthesis and Expression, Institute for Protein Research, Osaka University, Osaka 565-0871, Japan; 7 Department of Cardiovascular Medicine, Graduate School of Medical Science, Kyoto Prefectural University of Medicine, Kyoto 602-8566, Japan; 8 Division of Pharmacology, National Institute of Health Sciences, Kanagawa 210-9501, Japan; 9 Center for Infectious Disease Education and Research, Osaka University, Osaka 565-0871, Japan; 10 Laboratory of Nanofiber Technology, Gifu Pharmaceutical University, Gifu 501-1196, Japan

**Keywords:** ACE2 decoy, dry powder formulation, SARS-CoV-2, variants, escape mutations

## Abstract

The persistent threat of SARS-CoV-2 and the emergence of new variants has prompted the development of a novel, easily administered modality that can overcome viral mutations. The engineered ACE2 decoy shows neutralizing activity comparable to monoclonal antibodies and is broadly effective against SARS-CoV-2 variants and ACE2-utilizing sarbecoviruses. In addition to intravenous administration, this decoy has shown antiviral efficacy through nebulized aerosol inhalation in murine and primate models, offering a dose-sparing advantage. Clinically, dry powder formulation is ideal for convenience and storage but poses challenges for protein biologics. This study developed a freeze-dried spray formulation of the ACE2 decoy for inhalation. The trehalose and leucine-based excipient maintained neutralizing activity and prevented aggregate formation. The dry powder showed aerodynamic distribution from bronchi to alveoli, aiding protection against SARS-CoV-2 infections. Neutralizing activity, structural stability, and powder dispersibility were preserved after 6 months of storage. In a mouse model of SARS-CoV-2 infection, significant reductions in viral replication and lung pathology were observed with intratracheal administration 24 h post-infection. The ACE2 decoy retained activity against recent JN.1 and current KP.3 strains, confirming its robust efficacy against viral mutations. This ACE2 decoy powder inhalant is a self-administered, next-generation treatment addressing the ongoing immune-evading evolution of SARS-CoV-2.

## Graphical abstract

**Figure:**
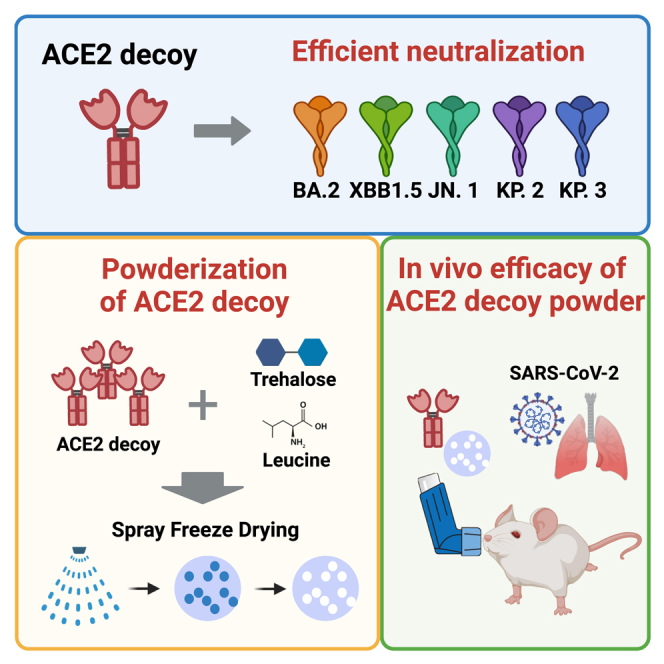

## Teaser

The engineered ACE2 decoy, administered via inhalation, neutralizes SARS-CoV-2 with broad-spectrum efficacy against most variants and even ACE2-using sarbecoviruses. Developed as a dry powder, it maintains neutralizing activity and structural integrity, reducing viral replication in infected mice. This inhalant addresses the evolving threat of SARS-CoV-2 with ease of self-administration.

## Introduction

During the COVID-19 pandemic, vaccines were developed at an unprecedented speed and played a central role in preventing SARS-CoV-2 infection and disease progression.^1^^,^^2^^,^^3^ However, the virus has acquired immune escape properties through mutations, and there are still cases of hospitalization and death due to SARS-CoV-2 infection. The elderly population and immunocompromised patients are particularly susceptible due to inadequate immunity provided by vaccines and thus require antiviral drugs to combat COVID-19.^4^ One therapeutic strategy is the inhibition of virus binding to the entry receptor ACE2.^5^^,^^6^ In line with this concept, monoclonal antibodies were developed early in the pandemic and numerous studies have confirmed their therapeutic efficacy in preventing hospitalization, disease severity, and death.^7^ However, continuous viral mutation and sequential replacement with dominant variants compromised the efficacy of monoclonal antibodies.^8^ The Omicron variant emerged with more than 30 mutations in the spike and exhibited escape from most monoclonal antibodies in clinical use.^9^^,^^10^^,^^11^ BA.2.86 is another variant with substantial antigenic drift and exhibits complete escape from all approved antibodies including sotrovimab, the only one that retained modest efficacy against XBB lineages.^12^ These facts reinforce the need for a therapeutic strategy to overcome viral mutation.

Receptor decoys are another modality that have a similar mode of action to monoclonal antibodies, whereby decoys bind to the receptor binding domain (RBD) of the spike and prevent the SARS-CoV-2 spike from binding to the host cell entry receptor. In contrast to monoclonal antibodies, receptor decoys have the potential to resist viral escape. If viruses acquire mutations that evade receptor decoys, the viruses will fail to bind the host cell receptor and lose infectivity.^13^ Previously, we developed the ACE2 decoy consisting of human immunoglobulin (Ig)G1-Fc and ACE2 with mutations that increase affinity and delete enzymatic activity.^14^ This engineered ACE2 decoy has similar neutralization efficacy to monoclonal antibodies and the advantage of covering all major variants.^14^^,^^15^^,^^16^ In addition, the engineered ACE2 was confirmed to have antiviral effects in rodent and monkey models of COVID-19 by either intravenous administration or nebulizer inhalation.^15^

ACE2 is expressed in the respiratory epithelium with particularly high levels on alveolar epithelial type II cells. SARS-CoV-2 primarily infects the upper airway and can spread to the alveoli, resulting in pneumonia and acute respiratory distress syndrome in severe cases.^17^ Therapeutic antibodies were approved for intravenous or intramuscular injections, but administration by these methods is inefficient for targeting the airway lumen, where antibodies need to cross the epithelium. In contrast, inhalation effectively achieves optimal drug concentration at local sites of infection, leading to dose-sparing benefits.^15^^,^^18^^,^^19^^,^^20^^,^^21^ Moreover, it is noninvasive and convenient for self-administration on an outpatient basis or at home, which is important for enabling early administration after clinical infection and, in turn, effective treatment.

A nebulizer and a dry powder inhaler (DPI) can be used for the inhalation of biologics. However, there are concerns about using nebulizers to apply for protein biologics. Nebulization with pressure or vibration and formulation as an aqueous solution or suspension induces the physical instability of protein including aggregation, denaturation, and hydrolysis.^22^ In addition, it takes time to prepare and administer with special equipment and electrical power. From a safety perspective, nebulized aerosol therapy poses a potential risk of fugitive emissions during therapy due to the generation of aerosols and droplets as a source of respiratory pathogens.^23^ Conversely, an inhalable dry powder formulation can preserve the native structure of proteins and extend product shelf life,^24^ and is safe and available with a portable device, which is ideal for clinical use.

In this study, we aimed to investigate the formulation of ACE2 decoy dry powder for clinical inhaler application with spray freeze-drying (SFD). The optimal excipient achieved long-term stability and aerodynamic properties broadly spreading to both bronchi and alveoli. Intratracheal administration in mice showed whole lung distribution and therapeutic efficiency in the SARS-CoV-2 MA10 infection model. Moreover, the engineered ACE2 retained neutralizing activity against BA.2.86/JN.1 and the currently dominant KP.2/KP.3, proving a valid hypothesis for all mutant strains.

## Results

### The engineered ACE2 decoy preserves neutralization against BA.2.86 subvariants

**Figure 1:**
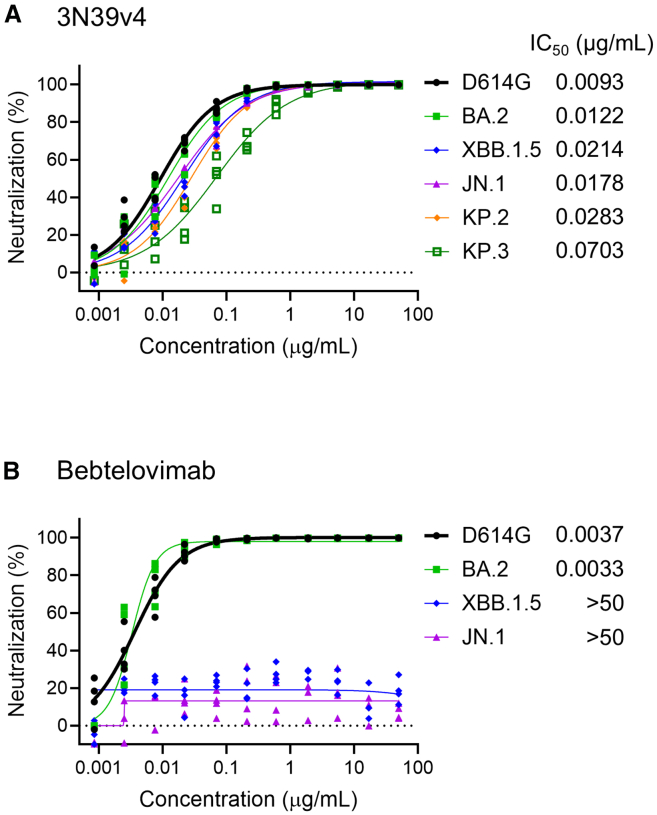
Engineered ACE2 decoy maintains neutralization efficacy against Omicron subvariants (A and B) Neutralization activity of 3N39v4 (A) and bebtelovimab (B) against Omicron subvariants was evaluated using the lentivirus-based pseudovirus system in 293T/ACE2 cells (*n* = 4).

As previously reported, our engineered ACE2 decoy, 3N39v4-Fc, has been demonstrated to be effective against Omicron subvariants, including XBB and BQ.1.^15^ Following the global dominance of the XBB lineage, the BA.2.86 lineage emerged with a long branch of evolution, comprising over 30 mutations in the spike region in comparison with BA.2.^12^ The BA.2.86 subvariant, designated JN.1, has emerged as a dominant variant, outcompeting the previously prevalent XBB subvariants. Currently, the KP.3 subvariant is dominant.^25^ We thus investigated the neutralizing activity of 3N39v4-Fc against BA.2.86 subvariants using a pseudotyped lentivirus. No apparent reduction in neutralization was observed for these subvariants, except for a slight weakness against KP.3 (Figure 1A). In contrast, bebtelovimab, the only approved monoclonal antibody that retained intact efficacy against BA.2 and BA.5,^26^^,^^27^ exhibited a complete loss of neutralizing efficiency against JN.1 (Figure 1B). The emergence of two major antigenic drift variants, BA.1 and BA.2.86, underscores the superiority of the ACE2 decoy, which can overcome viral mutations.

### The excipient is optimized for spray freeze-drying of the engineered ACE2 decoy

SFD is a technique that involves atomizing a liquid to produce smaller particles and freeze-drying to yield dry powders of controlled size and improved stability, making SFD suitable for drying thermally sensitive protein biologics for inhalation.^28^ However, SFD induces stresses such as dehydration, cold denaturation, and ice crystallization. It is therefore essential to use appropriate excipients to maintain stability.^29^ Sugars can protect proteins from denaturation and subsequent aggregation through vitrification and water replacement. This protective effect is often indispensable for maintaining biological activity during the process of SFD. We first compared the separate effects of trehalose, lactose, maltodextrin, α-cyclodextrin, β-cyclodextrin, hydroxypropyl β-CD (HPβCD), mannitol, and hydrophobic amino acids including leucine and phenylalanine on the stability and neutralizing function of the SFD ACE2 decoy. Lactose has a long and proven history in the formulation of dry powder inhalers.^30^ Trehalose is also widely used and superior at stabilizing proteins to other disaccharides because of its ability to form hydrogen-bonded networks with proteins and very low reactivity.^29^ In standard freeze-drying, trehalose and lactose preserved the protein structure and neutralizing activity of the ACE2 decoy. However, both trehalose and lactose have a highly absorbent nature, and their particles were unstable during the process of SFD (Table S1; Figure S1A). Cyclodextrins, in particular, HPβCD are promising excipients for protein stabilization and inhalable particles for pulmonary delivery^31^ through additional mechanisms such as direct binding or surface activity.^32^ For the ACE2 decoy excipients, α- and β-cyclodextrins resulted in insoluble powders. Instead, HPβCD powder was soluble and provided intact neutralizing activity, but was insufficient to prevent protein aggregation (Table S1; Figure S1A). Mannitol is also one of the most applicable excipients in drying methods and maltodextrin is added to increase protein stability.^33^ Neither alone prevented ACE2 decoy aggregation. Leucine and phenylalanine are hydrophobic amino acids and are used to protect spray-dried disaccharide formulations against moisture-induced changes.^34^ Powder with leucine alone was insoluble, and phenylalanine alone failed to maintain stability and activity (Table S1; Figure S1A).

Next, we focused on the highly protective effect of trehalose and lactose on the functional and structural states of ACE2 decoy in the traditional freeze-drying method (conditions 10 and 11 in Table S1). Since trehalose and lactose alone were unable to maintain the dry state in the SFD method (conditions 1 and 2 in Table S1), we decided to add leucine as a moisture-resistant excipient.^35^ A total of five conditions were investigated for each disaccharide, including the mixing ratio (w/w) of ACE2 decoy: trehalose/lactose: leucine of 5%:71.2%:23.8% (formulation “T/L1”), 5%:57%:38% (formulation “T/L2”), 5%:23.8%:71.2% (formulation “T/L3”), 20%:48%:32% (formulation “T/L4”), and 20%:20%:60% (formulation “T/L5”) (Table 1). In general, conditions with lower leucine concentration tended to preserve activity and induce less aggregation. However, SEM analysis of powders with 71.2% disaccharide formulation showed moisture-induced morphological alteration (Figure S1B). The 20 w/w% ACE2, 48 w/w% disaccharide and 32 w/w% leucine formulations (i.e., T4 and L4) exhibited the least formation of aggregation with almost intact neutralizing activity and moisture resistance. Therefore, we further evaluated the product of this mixing ratio.

**Table 1:** Dry powder formulations of the engineered ACE2 decoy produced via spray freeze-drying

Formulation	Ratio (%w/w)				IC_50_ (μg/mL)	Aggregate (%)
	ACE2	Trehalose	Lactose	Leucine		
T1	5	71.2	0	23.8	0.0387	15.46
T2	5	57	0	38	0.0609	28.58
T3	5	23.8	0	71.2	0.1209	19.39
T4	20	48	0	32	0.0407	11.30
T5	20	20	0	60	0.0339	18.26
L1	5	0	71.2	23.8	0.0326	16.41
L2	5	0	57	38	0.0589	34.93
L3	5	0	23.8	71.2	0.1968	33.66
L4	20	0	48	32	0.0299	14.00
L5	20	0	20	60	0.0460	35.91

The mixed formulations were prepared by blending the ACE2 decoy with disaccharides and leucine at a specific ratio (w/w). Neutralizing activity was evaluated against the ancestral SARS-CoV-2 pseudovirus, and the IC_50_ value was determined. The aggregation states were assessed by using size-exclusion chromatography (SEC) profiles, where % aggregation values were calculated from the integrated peak area table as described in the materials and methods section.

### The engineered ACE2 decoy dry powder diffusely distributes throughout the lungs

**Figure 2:**
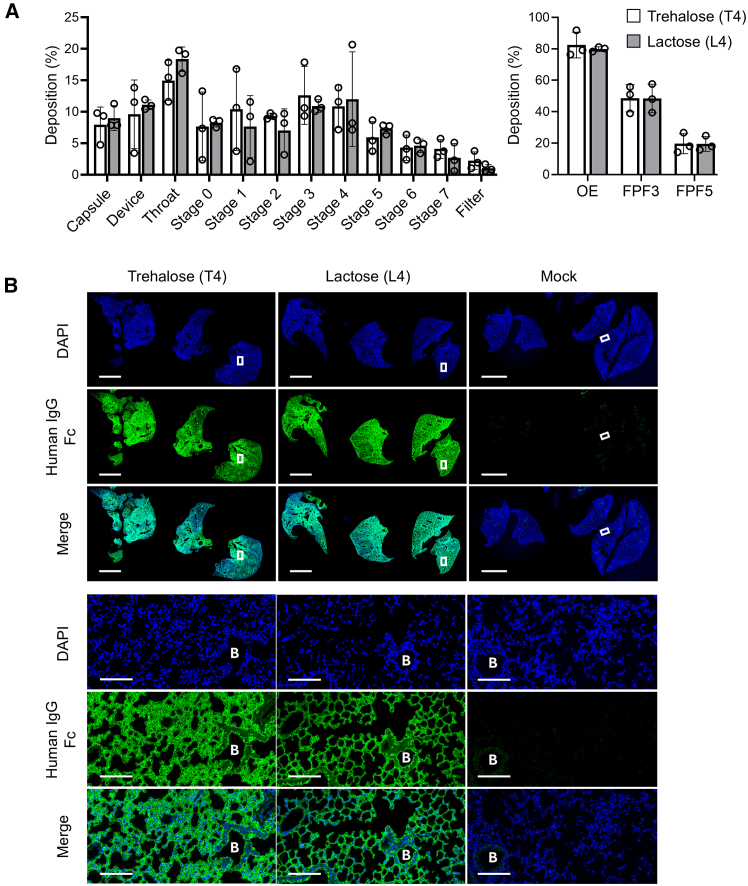
The ACE2 decoy dry powder exhibits a diffused deposition pattern at both the bronchiole and alveoli (A) Aerodynamic particle distribution of the ACE2 SFD powder made under T4 or L4 conditions in a multistage impactor. OE, output efficiency; FPF, fine particle fraction. Data are represented as mean ± SEM of *n* = 3. (B) Immunohistochemistry of ACE2 decoy in mouse lung after intratracheal administration. Scale bars, 2.0 mm in upper panels and 100 μm in lower panels. B indicates bronchiole.

The *in vitro* respirability of the SFD ACE2 decoy aerosol was extensively investigated with a multistage impactor using a powder mass of 5 mg, corresponding to 1 mg protein. The emitted rate of ACE2 decoy powder was 85.8% in trehalose (condition T4) and 77.7% in lactose (condition L4), indicating low loss of powder adhering to the inhaler chamber and mouthpiece. The distribution of the powder in the impactor was characterized by evenly depositing from stage 0–7, in which fine particle fraction3 (FPF3) and FPF5 values were 48.0% and 19.1% in the T4 sample and 44.2% and 17.5% in the L4 sample, respectively, indicating tracheal to peripheral pulmonary distribution (Figure 2A). Since the expression of ACE2 is diffusely distributed from nasal and oral mucosa, both upper and lower airway ciliated and secretory epithelial cells to alveolar epithelial cells,^36^ these aerodynamic characteristics are suitable for covering the regions that express ACE2. Next, the *in vivo* distribution of SFD ACE2 decoy aerosol was evaluated in mice by intratracheal administration of 0.3 mg powder, corresponding to 0.06 mg protein. Immunostaining of human IgG1-Fc, the component of ACE2 decoy, showed homogeneous distribution, and no enrichment in the bronchiolar tissue. Magnified images revealed comparable deposition in the alveoli and bronchioles (Figure 2B). These results were consistent with those of *in vitro* assessments with an impactor and showed promise for *in vivo* antiviral efficacy.

### The engineered ACE2 decoy in the SFD powder is stable over long periods

**Figure 3:**
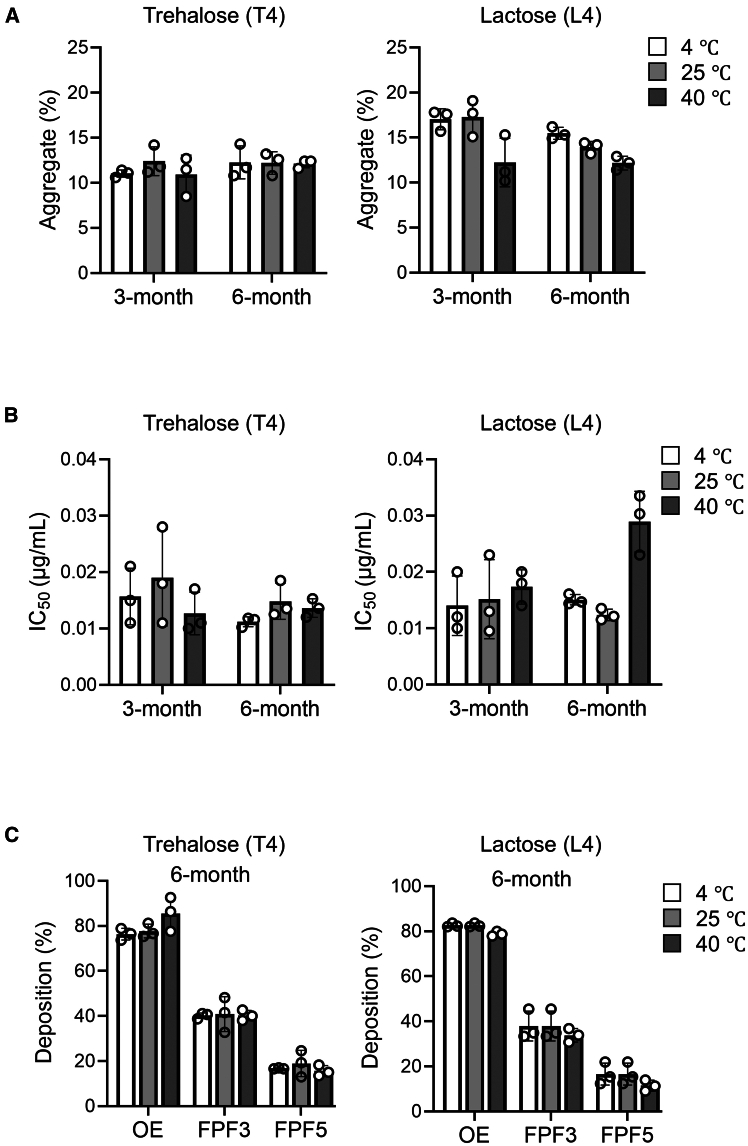
Long-term structural and functional stability of the ACE2 decoy dry powder (A) SFD powders (T4 and L4 excipient formulation, Table 1) were stored for up to 6 months in three different conditions: cold (4°C), ambient (25°C with humidity of 30%–60% RH), and accelerated (40°C with humidity of 75% RH) conditions. The proportion of aggregates was assessed by using size-exclusion chromatography (SEC) as described in the materials and methods section at 3 and 6 months. (B) Neutralizing activity was evaluated against the ancestral SARS-CoV-2 pseudovirus, and the IC_50_ value was determined at 3 and 6 months. (C) Aerodynamic particle distribution of the ACE2 decoy dry powder was evaluated by a multistage impactor at 6 months. Raw data are presented in Figure S2. Data are represented as mean ± SEM of *n* = 3 (A to C).

Another potential benefit of SFD biologics is the extended shelf life especially in non-refrigerated conditions. To maintain superior aerodynamics, however, the powder needs to remain dispersed. In addition, the protein’s biophysical, chemical, and functional integrity must be preserved even without cold chain storage. To address these issues, we first analyzed the size-exclusion chromatography (SEC) profiles of the SFD ACE2 decoy samples stored over 6 months. The ACE2 decoy powder (conditions T4 and L4, Table 1) was stored under three different conditions: cold (4°C), ambient (25°C with humidity of 30%–60% relative humidity [RH]), and accelerated (40°C with humidity of 75% RH) conditions. The SEC analysis showed that original fresh ACE2 decoy sample already contained up to 8% multimeric species (see materials and methods), which was slightly increased to 10%–15% upon the SFD treatment (Table 1). However, no increase in the amount of aggregated species were observed with samples stored 3 or 6 months under any of the three conditions (Figures 3A and S2A). We also performed dynamic light scattering (DLS) analysis on the T4 and L4 samples stored for 1 year at ambient condition. Again, SFD process and long-term storage increased aggregation only slightly, up to less than 1% (Figure S2B). Additionally, the SDS-PAGE analysis did not reveal the appearance of either slow-migrating bands corresponding to covalently multimerized species or fast-migrating bands corresponding to degraded species, indicating that there were no gross chemical alterations during this storage (Figure S2C). The pseudovirus neutralizing activity remained unaltered for 6 months in the trehalose-based excipient (T4), whereas the lactose-based excipient (L4) exhibited modest impairment after storage at 40°C for 6 months (Figures 3B and S3A). The aerodynamic particle distribution was confirmed to be stable over a 6-month period. The emitted rate, FPF3, and FPF5 exhibited similarities to the baseline across all three distinct conditions (Figures 2A, 3C, and S3B). These data demonstrate the superior functional stability of the trehalose-based excipient over time.

### Intratracheal administration of the engineered ACE2 decoy powder efficiently treats SARS-CoV-2 infection in rodents

**Figure 4:**
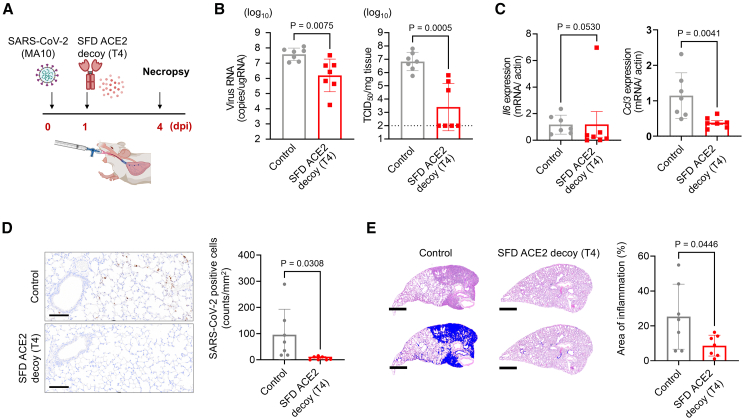
Intratracheal administration of ACE2 decoy dry powder is efficacious in a mouse model of SARS-CoV-2 infection (A) Experimental design to establish a mouse model of SARS-CoV-2 infection using mouse-adapted SARS-CoV-2 (MA10). Balb/c mice were infected intranasally (i.n.) with MA10 at 1.0 × 10^4^ TCID_50_ (in 20 μL) and ACE2 decoy powder made by T4 formulation SFD was administered 24 h after infection. (B) Viral RNA and infectious virus titers in the lungs of control and SFD ACE2 decoy-treated mice at 4 days post-infection (dpi). (C) mRNA expression of Il6 and Ccl3 in the lungs of control and SFD ACE2 decoy-treated mice at 4 dpi. (D) Immunohistochemistry of SARS-CoV-2 antigen in the lungs of control and SFD ACE2 decoy-treated mice at 4 dpi. Antigen-positive cells are quantified (left). Scale bars, 100 μm. (E) Lung tissue sections of the mice in the control and treatment groups were stained with hematoxylin and eosin (H&E). H&E-stained sections (top row) and H&E-stained sections with blue highlights on the inflammatory area (bottom row) are shown. Scale bars, 800 μm. The ratio of the inflammatory area to the total lung area was calculated. Data are presented as mean ± SEM of *n* = 7 each group. *p* values were determined using Mann-Whitney *U* test (B to E).

Given that trehalose proved more efficacious than lactose in maintaining protein structure and long-term functionality, we employed a trehalose-based excipient (i.e., formulation T4) to confirm the antiviral effects of the optimized SFD powder of the engineered ACE2 decoy. BALB/c mice were intranasally infected with 1 × 10^4^ tissue culture 50% infectious dose (TCID_50_) of mouse-adapted SARS-CoV-2 (SARS-CoV-2 MA10),^37^ and 0.3 mg of dry powder, corresponding to 0.06 mg of ACE2 decoy, or excipient-only control powder was administered through the inhaled route 24 h after inoculation (Figure 4A). The lungs were harvested 4 days post-infection, and the therapeutic effect of the SFD ACE2 decoy was evaluated. Viral RNA, viral titers, and the expression of inflammatory cytokine Ccl3 were significantly suppressed, and Il6 expression was relatively inhibited in the treated mice (Figures 4B and 4C). Additionally, histopathological analyses were conducted on infected mice. Immunostaining of SARS-CoV-2 revealed a near-complete suppression of the viral antigen in the ACE2 decoy-treated lungs (Figure 4D). The excipient-only powder treatment resulted in the development of severe pneumonia, which was characterized by the presence of widespread inflammatory cell infiltration and alveolar hemorrhage. In contrast, the inflammatory area was significantly reduced in the SFD ACE2 decoy treatment group (Figure 4E).

The cardiotoxicity of the SFD ACE2 decoy was evaluated in human induced pluripotent stem cell-derived cardiomyocytes (hiPS-CMs) because ACE2 plays an important role in the cardiovascular system, including regulation of blood pressure and cardiac function,^38^ although the enzymatic activity of ACE decoys is inactivated.^14^ In addition, the development of numerous drug candidates has been delayed or canceled as a consequence of their unexpected cardiotoxicity, including the Torsades de pointes (TdP) risk associated with QT interval prolongation.^39^ The administration of trehalose or lactose-based SFD ACE2 decoys (T4 and L4) did not result in the prolongation of field potential (FP) duration or the occurrence of early afterdepolarization, which is a critical trigger for the initiation of TdP (Figure S4A). Furthermore, treatment with the SFD ACE2 decoy did not affect beating rate, cardiac contractility, or relaxation, as determined by imaging-based motion analysis (Figures S4B–S4D).

## Discussion

The engineered ACE2 decoy, composed of a high-affinity and peptidase-dead ACE2 mutant and IgG1-Fc, possesses neutralizing activity comparable to therapeutic antibodies and broad-spectrum efficacy against SARS-CoV-2 variants, offering a competitive advantage over existing treatments. Consistent with the concept that mutant viruses that evade the ACE2 decoy are unable to bind cell surface ACE2 and lose viral fitness, the efficacy of the decoy has been confirmed from the ancestral strain to XBB subvariants.^14^^,^^15^^,^^16^ However, the next major variant, BA.2.86, emerged with more than 30 additional mutations in the spike. As a result, all approved antibodies, including sotrovimab, showed a complete loss of efficacy against this variant.^25^ Notwithstanding the considerable antigenic drift, this study confirmed that the ACE2 decoy retained functional neutralizing capacity against BA.2.86 and its subvariants, including the currently circulating KP.3. This observation supports the concept that the decoy is effective for all variants of SARS-CoV-2.

Compared with intravenous or intramuscular administration, respiratory delivery, which directly reaches the infected area of the virus, provides a more effective antiviral effect. Pharmacodynamic analysis showed that when injected intravenously, the level of monoclonal antibodies in the mucosal compartment was 200–500 times lower than that observed in the serum.^40^ In addition, in a murine model of SARS-CoV-2 infection, inhalation of nebulized aerosols of ACE2 decoys and monoclonal antibodies showed therapeutic efficacy comparable to intravenous injection at a 20–50 times lower dose.^15^^,^^18^^,^^19^ The superiority of respiratory delivery in antiviral efficacy has also been confirmed for influenza virus^41^^,^^42^ and respiratory syncytial virus.^43^ Nebulizers and DPIs are commonly used to inhale medications. Nebulizers utilize a liquid solution of the drug to generate aerosol droplets. While not requiring a specialized formulation, the long-term storage of proteins in liquid solutions causes unfavorable changes, including deamination, aggregation, and hydrolysis.^22^ DPIs are propellant-free devices that deliver dry powder aerosols to the lungs and have the advantage of a longer shelf life without refrigeration. However, protein biologic solutions have a tendency to aggregate during spray drying and require suitable excipients to prevent this problem.^44^

In this study, the disaccharides trehalose and lactose were selected as excipients for DPIs, and leucine was chosen as a moisture-resistant excipient. Upon spray freeze-drying aqueous solutions of lactose and trehalose, the resulting individual products are observed to be in an amorphous state. It is established that disaccharides in an amorphous state contribute to the stabilization of proteins in a solid state.^45^^,^^46^ The disaccharide-containing SFD dry powders demonstrated the maintenance of neutralizing activity of the ACE2 decoy (Table S1). However, the SFD powders consisting solely of disaccharides possess a large specific surface area due to their high porosity, which resulted in moisture absorption even when stored in a silica gel container maintained at 30% RH^47^ (Figure S1; Table S1). Accordingly, the present study aimed to enhance the moisture absorption resistance of SFD powders containing disaccharides by incorporating leucine. Leucine is utilized as a pharmaceutical additive, and it exhibits physicochemical properties that are highly resistant to moisture, absorbing minimal moisture in a typical indoor environment.^48^ However, the SFD dry powder consisting solely of leucine proved difficult to redissolve, indicating the potential for aggregation of the ACE2 decoy (Table S1). Therefore, the composition ratio of disaccharides and leucine in SFD dry powders was optimized, and the optimal ratio of disaccharides and leucine was determined in terms of water solubility, protein stability, moisture absorption resistance, and aerosol performance (Table 1). The dry powders with a mixing ratio of disaccharides to leucine of 3:1 (T1 and L1) demonstrated favorable half maximal inhibitory concentration (IC_50_) values and stability. However, they exhibited a gradual absorption of moisture (Figure S1). On the other hand, dry powders with a mixing ratio of 1:3 (T3, T5, L3, and L5) demonstrated an increase in aggregation rate. It is noteworthy that the IC_50_ values and stability were markedly enhanced when the ACE2 decoy composition was increased to 20% (T4 and L4), although a reduction in the protein composition ratio generally contributes to the stabilization of the protein in the dry powder.^47^

The *in vitro* aerosol performance of SFD ACE2 decoy composed of 48 w/w% disaccharide and 32 w/w% leucine (T4 and L4) was fascinating (Figure 2A). Considering that the output efficiency (OE) values of DPI products with a capsule on the market range from 55% to 80%, the SFD dry powders in this study have already reached a level that is conducive to practical application in terms of aerosol performance.^49^ For effective drug delivery to the lung, the aerodynamic particle diameter, calculated from the geometric diameter and density, must be reduced to a single micron.^50^ As a geometric diameter, single-micron particles cause adhesion forces between particles to predominate over gravity, resulting in agglomeration and consequently a reduction in drug release and delivery to the lungs. SFD powders have high porosity and low density, which effectively inhibit particle agglomeration and ensure aerosol performance suitable for DPIs.^51^ This formulation concept has been successfully commercialized in the form of the Tobi Podhaler (PulmoSphere) powder for inhalation.^52^ The aerosol performance of the powder remained unaltered when stored in silica gel with the addition of leucine.^53^ Leucine may also help prevent the formation of solid bridges between particles by functioning as a surfactant and reducing particle adhesion and aggregation.^51^ The presence of leucine on the powder surface protects amorphous solids from moisture, inhibits the crystallization of excipients added to the formulation, and may enhance the stability of amorphous powders.^54^ In addition, although lactose is a reducing sugar, raising concerns about the potential for a Maillard reaction with the ACE2 decoy,^55^ no evidence of such a reaction was observed in this study (Figure 3).

The dry powder formulation of the engineered ACE2 decoy allows for self-administration at home without the need for special equipment or medical personnel, similar to small molecule compounds. Moreover, the high efficiency of the dry powder inhalation allows for a reduction in the required dosage, thereby conserving medical resources and facilitating the expansion of deployments. The ACE2 decoy is effective against the current KP.3 and is expected to be resilient to future SARS-CoV-2 evolution and even in the event of a future spillover from a zoonotic reservoir.^16^ Overall, the ACE2 decoy dry powder is anticipated to become a next-generation medicine for SARS-CoV-2 infection, and the receptor decoy dry powder formulation may be applied to other respiratory viral infections.^56^^,^^57^

## Materials and methods

### Study design

All rodent experiments on SARS-CoV-2 were performed in biosafety level 3 (ABSL3) facilities at the Research Institute for Microbial Diseases, Osaka University (Osaka, Japan). The experimental protocols were approved by the Institutional Committee of Laboratory Animal Experimentation of the Research Institute for Microbial Diseases, Osaka University (approval number R02-08-0). All efforts were made to minimize animal suffering and to reduce the number of animals used in the experiments.

### Plasmid generation

A plasmid encoding the SARS-CoV-2 Spike was obtained from Addgene (#145032). Mutations for Omicron subvariants and the ΔC19 deletion (with 19 amino acids deleted from the C terminus) were cloned into the KpnI-XhoI sites of the pcDNA4TO (Thermo Fisher Scientific).^14^ ACE2 decoy (3N39v4-Fc) was cloned into the HindIII-BamHI sites of the pCMV plasmid with the Igκ signal sequence (https://benchling.com/s/seq-0leUpBlmMGPehwBNHAVD?m=slm-tQsnbXex64fLulMlW4ol). psPAX2-IN/HiBiT and pLenti Firefly were used previously.^14^^,^^58^ All cloning experiments were performed using NEBuilder HiFi DNA Assembly (New England Biolabs), and plasmid sequences were confirmed using a DNA sequencing service (Macrogen Japan Corp).

### Cell culture

Lenti-X 293T cells (Takara Bio) and their derivative, 293T/ACE2 cells, were cultured at 37°C in 5% CO_2_ in Dulbecco’s modified Eagle’s medium (DMEM, WAKO) containing 10% fetal bovine serum (FBS) (Gibco) and penicillin/streptomycin (P/S) (100 U/mL, Invitrogen). Vero E6/TMPRSS2 cells were a gift from the National Institutes of Biomedical Innovation, Health and Nutrition (Japan) and were cultured at 37°C in 5% CO_2_ in DMEM (WAKO) containing 5% FBS (Gibco) and P/S (100 U/mL, Invitrogen). All cell lines were routinely tested for mycoplasma contamination.

### Viruses

Mouse-adapted SARS-CoV-2^37^ was generated using circular polymerase extension reaction (CPER), as previously described.^59^^,^^60^ This virus was propagated in Vero/TMPRSS2 and VeroE6/TMPRSS2 cells. Viral titers were determined by tissue culture 50% infectious dose (TCID_50_) methods using VeroE6/TMPRSS2 cells. Briefly, 1 day prior to infection, VeroE6/TMPRSS2 cells (10,000 cells) were seeded into a 96-well plate. Serially diluted samples were inoculated into the cells and incubated at 37°C for 4 days. The cells were observed under a microscope to assess the appearance of cytopathic effects. The TCID_50_/mL value was calculated according to the Reed-Muench method.^61^

Pseudotyped reporter virus assays were conducted as previously described.^14^ Spike protein-expressing pseudoviruses with a luciferase reporter gene were prepared by transfecting plasmids (pcDNA4TO SARS-CoV-2 Spike, psPAX2-IN/HiBiT, and pLenti Firefly) into LentiX-293T cells using Lipofectamine 3000 (Invitrogen). After 48 h, supernatants were collected, filtered through a 0.45-μm low protein-binding filter (SFCA), and frozen at −80 °C.

### Protein synthesis and purification

Monoclonal antibodies were expressed using the Expi293F cell expression system (Thermo Fisher Scientific) according to the manufacturer’s protocol. Secreted proteins were purified using rProtein A Sepharose Fast Flow (Cytiva). Fractions containing target proteins were pooled and dialyzed against PBS.^14^

Engineered ACE2 protein synthesis and purification were conducted in Thermo Fisher Scientific. Briefly, 3N39v4-Fc was expressed using the Expi293F cell expression system at a scale of 20 L with 20 mg of plasmid. Secreted proteins underwent protein A purification with MabSelect SuRe (Cytiva). Fractions containing target proteins were further purified through gel filtration using Superdex 200 pg (Cytiva). Fractions containing target proteins were pooled and evaluated by SDS-PAGE and SEC. The resulting yield of ACE2 decoy protein was 1,185 mg.

### Preparation of solution and dry powders with ACE2 decoy

The powder excipients employed in this study were trehalose dihydrate (WAKO), lactose monohydrate (WAKO), L-leucine (WAKO), α-cyclodextrin (Nacalai Tesque), β-cyclodextrin (WAKO), hydroxypropyl β-cyclodextrin (Junsei Chemical), D(−)-mannitol (WAKO), maltodextrin (Sigma-Aldrich), and L-phenylalanine (Sigma-Aldrich). The preparation of solutions and dry powders with ACE2 decoy is detailed in Tables 1 and S1. As previously outlined in the extant literature, the preparation procedure for dry powders by SFD is as follows.^62^ All components were dissolved in water and thoroughly mixed in test tubes. Each sample solution (50 mg/5 mL) was atomized into liquid nitrogen with a 0.4-mm two-fluid nozzle positioned approximately 15 cm above the surface of the liquid nitrogen at a flow rate of 5 mL/min and an atomizing pressure of 150 kPa, thereby generating frozen droplets. The frozen droplets were subsequently transferred into a freeze dryer (DRC-1100 and FDL-2000, Tokyo Rikakikai), which had been precooled to a shelf temperature of −40°C. Once the liquid nitrogen had evaporated completely, the freeze-drying process was initiated. This involved the application of a vacuum level of 5 Pa at a shelf temperature of 10°C for a period of 24 h, resulting in the formation of SFD powders. The individual dry powder samples, prepared in triplicate, were subsequently combined into a single glass vial.

### Long-term storage

All mixed powders were then distributed in equal proportions into seven test tubes, which were then sealed with KimWipes and rubber bands. The test tubes were stored in six airtight containers with silica gel, which were sealed and stored in a controlled environment. The six containers were placed in duplicate under three storage environments: a refrigerator (4°C; MPR-414FS, Panasonic), a general indoor environment (25°C; 20°C–30°C and 30%–60% RH), and an accelerated condition (40°C/75% RH; HPAV-48-20, ISUZU). The duplicate airtight containers were stored in each storage environment for a period of 3 and 6 months, respectively.

### Morphological observations of dry powders

The morphological properties of the dry powders were examined using a scanning electron microscope (SEM, JSM- 6510LV, JEOL).^63^ The dry powders were dispersed on a specimen mount with double-sided carbon tape (8 mm × 8 mm) using a spatula. The samples were coated with gold for 1 min using a coating machine (DII-29010SCTR Smart Coater, JEOL) and imaged at an accelerating voltage of 10 kV.

### *In vitro* aerosol performance of dry powders

The *in vitro* aerosol performance of the dry powders was evaluated using an eight-stage Andersen cascade impactor (ACI; AN-200; Tokyo Dylec). The conditions for evaluation were previously described.^62^ To prevent powder bouncing caused by the air stream of inspiration, the metal plates of the stages were coated with glycerin. A No. 2 hydroxypropyl methylcellulose hard capsule (Shionogi Qualicaps), containing approximately 5 mg of each dry powder was placed in a dry powder inhaler (Jethaler reverse type; Tokico System Solutions). Inspiration was conducted at a flow rate of 28.3 L/min for a duration of 8.5 s, which equates to approximately 4 L of inspiration volume, using a vacuum pump. The powder deposited on each stage (capsule, device, throat, stages, and filter) was dissolved in 5 mL of distilled water. The mass of the deposited dry powder on each stage was estimated based on the protein (ACE2 decoy) concentration, which was determined using the bicinchoninic acid (BCA) protein assay, according to the manufacturer’s instructions (BCA Protein Assay Kits, Thermo Scientific). Briefly, 100 μL of the dissolved powder from each stage was added to a test tube and mixed with 1 mL of the working reagent. Subsequently, the test tubes were incubated at 60°C for 30 min. After cooling the test tubes to room temperature, the absorbance at 562 nm was determined using a microvolume UV-Vis spectrophotometer (NanoDrop, Thermo Scientific). The inhalation characteristics were evaluated based on three inhalation indexes: output efficiency (OE) and fine particle fraction (FPF) 3 and FPF 5, which were calculated using the deposition of dry powder on each stage:

Deposition (%) = mass in each part/total mass × 100

OE (%) = mass in throat and lower parts/total mass × 100

FPF3 (%) = mass in stage 3 and lower parts/mass in throat and lower parts × 100

FPF5 (%) = mass in stage 5 and lower parts/mass in throat and lower parts × 100

OE represents the emission potential of the capsule and device. FPF3 and FPF5 signify the potential for delivery into the lung (the cutoff diameter in stage 3 was 4.7 μm) and the alveoli (the cutoff diameter in stage 5 was 2.1 μm), respectively.

### Biophysical characterization of SFD samples

The SFD ACE2 decoy samples prepared under various conditions were reconstituted by gently adding water to make ∼0.5 mg protein/mL solution and were left for 5 min without agitation. Any conditions that showed large visible precipitates were remarked as “insoluble” and omitted from further analysis. The apparently clear mixture was centrifuged at 10,000 × *g* for 10 min to remove any trace amount of insoluble materials, and the supernatant was subjected to SEC. Samples containing ∼50 μg of decoy protein were injected onto a Superdex 200 Increase 10/300GL column (Cytiva) equilibrated with 20 mM phosphate, 150 mM NaCl, pH 7.2 (PBS) at a flow rate of 1.0 mL/min in an AKTApure chromatography system (Cytiva). Protein elution was monitored by A280 and the ACE2 decoy monomer (although it is actually a dimer because of the Fc fusion) was mainly eluted at ∼10.3 mL peak. Any preceding peaks eluting at 8–10 mL (indicated by asterisk in Figure S2A) corresponded to higher molecular weight species. Each peak area value was obtained by the peak integration tool in the Evaluation module of UNICORN software (Version 6.4, Cytiva) and % aggregation values were calculated by dividing the combined peak areas for 8–10 mL with the sum of all peaks. The original sample contained 4%–8% aggregates even before the SFD process. Monodispersity of the ACE2 decoy after SFD procedure was also evaluated by dynamic light scattering (DLS) as follows. After reconstitution, protein samples (∼1 mg/mL) were transferred to a quartz microcuvette (3 mm light path, 9.65 mm center height, Hellma 105-251-005 QS) and analyzed on a Zetasizer μV (Malvern Panalytical). Analysis was repeated three times and averaged data are processed using Zetasizer software version 8.02 to derive distribution histograms. Samples were also analyzed by SDS-PAGE under reducing (i.e., in the presence of 20 mM DTT) and non-reducing conditions using 7.5% acrylamide gel (e-pagel, #2331830, ATTO Corporation) and stained with Coomassie Brilliant Blue.

### Pseudotyped lentivirus neutralization assay

293T/ACE2 cells were seeded at 10,000 cells/well in a 96-well plate. HiBit value-matched pseudovirus and a 3-fold dilution series of therapeutic agents were incubated for 1 h. This mixture was then administered to 293T/ACE2 cells. After 1 h of pre-incubation, the medium was changed, and cellular expression of the luciferase reporter indicating viral infection was determined 48 h after infection using the ONE-Glo Luciferase Assay System (Promega). Luminescence was measured using an Infinite F200 Pro System (Tecan).^15^

### Rodent models of SARS-CoV-2 infection

In the BALB/c mouse model, 10-week-old female BALB/c mice were purchased from SLC Japan or CLEA Japan. Mice were anesthetized by isoflurane and challenged with SARS-CoV-2 MA10 (1.0 × 10^4^ TCID_50_ in 20 μL) through the intranasal route. Then, 24 h post-infection, 0.3 mg of ACE2 decoy powder or excipient-only control was administered through intraperitoneal injection or inhalation as described below. At 4 days post-infection, the animals were euthanized, and their lungs were collected for qPCR and virus titration.

### Intratracheal administration in rodents

BALB/c mice were anesthetized by intraperitoneal administration of 10 μL/g body weight of a medetomidine-midazolam-butorphanol tartrate mixture; 0.75 mg/kg-medetomidine (ZENOAQ), 2 mg/kg midazolam (Maruishi-pharm), and 2.5 mg/kg butorphanol tartrate (Meiji Seika). Anesthetized animals were placed on the intubation stand (Natsume Seisakusho, KN-1014). Blunt forceps were used to displace the tongue for insertion of the intubation tube using a laryngoscope (Natsume Seisakusho, KN-1021). A cannula (polyethylene tube: total length of 4.0 cm, cut from indwelling feeding tube for infant [Atom, 43003]) was inserted into the trachea. Then, 0.3 mg of ACE2 decoy powder or excipient-only control, taken in a disposable tip, was dispersed using an administration device as indicated previously.^62^ In brief, a three-way stopcock and a 1-mL syringe containing 0.3 mL of air were connected to the disposable tip. Mice were anesthetized and orally intubated with the cannula connected to the disposable tip, three-way stopcock, and 1-mL syringe. By pressing the 1-mL syringe and opening the three-way stopcock, the dry powder was dispersed into the trachea. To ensure that no visible powder remained in the tip, air was instilled into the connected yellow tip several times. Atipamezole (ZENOAQ) was administered intraperitoneally to reverse the anesthesia.

### Histological analysis

From each lung, the left lower, right lower, left upper, and right upper lobes were dissected and routinely processed to prepare formalin-fixed paraffin-embedded tissue samples. The samples were cut into 2-μm-thick tissue sections and stained with hematoxylin and eosin (H&E). H&E-stained tissue sections were scanned using SLIDEVIEW VS200 (Olympus Life Science) to acquire whole-slide digital virtual slide images. QuPath was used to train the algorithm to classify the histologic pattern of the inflammatory area, and the ratio of the inflammatory area to the total lung tissue area was calculated. For immunohistochemical detection of SARS-CoV-2, 2-μm-thick sections were immersed in citrate buffer (pH 6.0) and heated for 20 min with a pressure cooker. Endogenous peroxidase was inactivated by immersion in 3% H_2_O_2_ in PBS. After treatment with 5% BSA in PBS for 30 min at room temperature, the sections were incubated with in-house rabbit anti-SARS-CoV-2 N antibody^64^ at 4°C overnight. Then, the slides were washed with PBS and incubated with Histofine Simple Stain MAX PO (R) (Nichirei Bioscience, Cat #424141, RRID: AB_3073750) at room temperature for 40 min. Positive signals were then visualized by peroxidase–diaminobenzidine reaction, and sections were counterstained with hematoxylin. The sections were scanned by SLIDEVIEW VS200 (Olympus Life Science) to acquire whole-slide digital virtual slide images. Positive cell densities were calculated with image analysis software QuPath. For detection of ACE2 decoy in the lung, 2-μm-thick sections were incubated with HRP-conjugated goat-anti-human IgG Fc fragment (Abcam, 1:200) after blocking with 5% BSA. Then, the signals were visualized with Opal520 fluorescent dye by the tyramide signal amplification system using Opal520 reagent pack (Akoya Biosciences). After staining with DAPI, the slides were scanned with PhenoImager Fusion (Akoya Biosciences).

### Quantitative PCR of *in vivo* samples

Total RNA from lung homogenates was isolated using ISOGENE II (NIPPON GENE). Quantitative PCR (qPCR) was performed using the Power SYBR Green RNA-to-CT 1-Step Kit (Thermo Fisher Scientific) on the QuantStudio3 Real-Time PCR (Thermo Fisher Scientific). Relative quantitation of target mRNA concentrations was determined using the 2-ΔΔCT method. The values were normalized to those of the housekeeping gene, β-actin. The following primers were used: *Actb*, 5′-TTGCTGACAGGATGCAGAAG-3′ and 5′-GTACTTGCGCTCAGGAGGAG-3′; 2019-nCoV_N2, 5′- AATTTTGGGGACCAGGAAC-3′ and 5′-TGGCAGCTGTGTAGGTCAAC-3′; *Il6* 5′-CCACTTCACAAGTCGGAGGCTTA-3′ and 5′-GCAAGTGCATCATCGTTGTTCATAC-3′, *Ccl3* 5′-AACCAGCAGCCTTTGCTCCC-3′ and 5′-GGTCTCTTTGGAGTCAGCGCA-3′.

### Field potential recordings

Fluid potentials (FPs) were recorded as previously described.^65^ Briefly, before measuring FPs, hiPS-CMs were incubated in a 5% CO_2_ incubator at 37 °C for more than 30 min after replacement with fresh medium. To stabilize the waveform, the probes were set on an MEA system (MED64, Alpha MED Scientific) and equilibrated in a humidified 5% CO_2_ atmosphere for at least 30 min. After treatment with ACE2 decoy powder, FP recordings were performed for 10 min. Vehicle- and baseline-corrected field potential duration corrected Fridericia’s formula (ΔΔFPDc) was calculated for ACE2 decoys.^66^

### Motion analysis

The motion of hiPSC-CMs was recorded using the SI8000 cell motion imaging system (Sony), as previously reported.^67^ Sequential phase-contrast images were obtained using a 10× objective at 150 frames per second and a resolution of 2,048 × 2,048 pixels at 150 Hz for 10 s at 37°C using the SI8000 cell motion imaging system (Sony). The recorded movie images were analyzed with a block-matching algorithm using an SI8000 analyzer.^68^

### Statistical analysis

Neutralization measurements were done in technical triplicates and relative luciferase units were converted to percent neutralization and plotted with a non-linear regression model to determine 50% inhibitory concentration (IC_50_) values using GraphPad Prism software (version 9.0.0). Comparisons between two groups were made with Mann-Whitney *U* tests. *P* values <0.05 were considered statistically significant.

## Data and code availability

All processed data supporting the findings of this study are available within the paper and its supplemental information. Unprocessed data are available from the corresponding authors upon reasonable request.

## Acknowledgments

We would like to thank K. Tokunaga (Department of Pathology, NIID) for the gift of the plasmid-coding psPAX2-IN/HiBiT, and Mika Olivia Rohlfing for polishing the language. This work was supported by 10.13039/100009619Japan Agency for Medical Research and Development (AMED), Research Program on Emerging and Re-emerging Infectious Diseases under 22fk0108523 (A.H., J.T., T.O., K.T., Y.S., and Y.K.) and by 10.13039/501100001691JSPS KAKENHI Grant Number 24K21951 (A.H.). Experimental schemes were illustrated by BioRender.

## Author contributions

A.H., T.O., and K.T. designed the research; T.I. and K.T. optimized the formulation of dry powder and evaluated aerodynamic particle distribution; K.N. and N.I. performed pseudovirus neutralization assay; J.T. performed size-exclusion chromatography, and SDS-PAGE; K.S. performed DLS experiments and analyzed the data.; T.S., Y.S., Y.I., and T.O. conducted authentic SARS-CoV-2 experiments in mice; Y.K. evaluated the toxicity in iPSC derived cardiomyocytes; S.M., T.O., K.T., and A.H. supervised the research; A.H. wrote the manuscript; all authors discussed the results and commented on the manuscript.

## Declaration of interests

A.H., S.M., J.T., and T.O. are inventors on a patent filed by Kyoto Prefectural University of Medicine and Osaka University for engineered ACE2 decoy.

## Supplemental information

Document S1. Figures S1–S4 and Table S1

Document S2. Article plus supplemental information
