# Supplementary material for: Engineered ACE2 decoy in dry powder form for inhalation: A novel therapy for SARS-CoV-2 variants
Source: Mol Ther Methods Clin Dev. 2025 Mar 31;33(2):101459. doi: 10.1016/j.omtm.2025.101459 (PMC12019485; doi:10.1016/j.omtm.2025.101459)
Supplement: Document S1. Figures S1–S4 and Table S1 [file mmc1.pdf]

OMTM, Volume *33*

Supplemental information

Engineered ACE2 decoy in dry powder form for inhalation: A novel therapy for SARS-CoV-2 variants

Takaaki Ito, Tatsuya Suzuki, Yusuke Sakai, Keisuke Nishioka, Yumi Itoh, Kentarou Sakamoto, Nariko Ikemura, Satoaki Matoba, Yasunari Kanda, Junichi Takagi, Toru Okamoto, Kohei Tahara, and Atsushi Hoshino

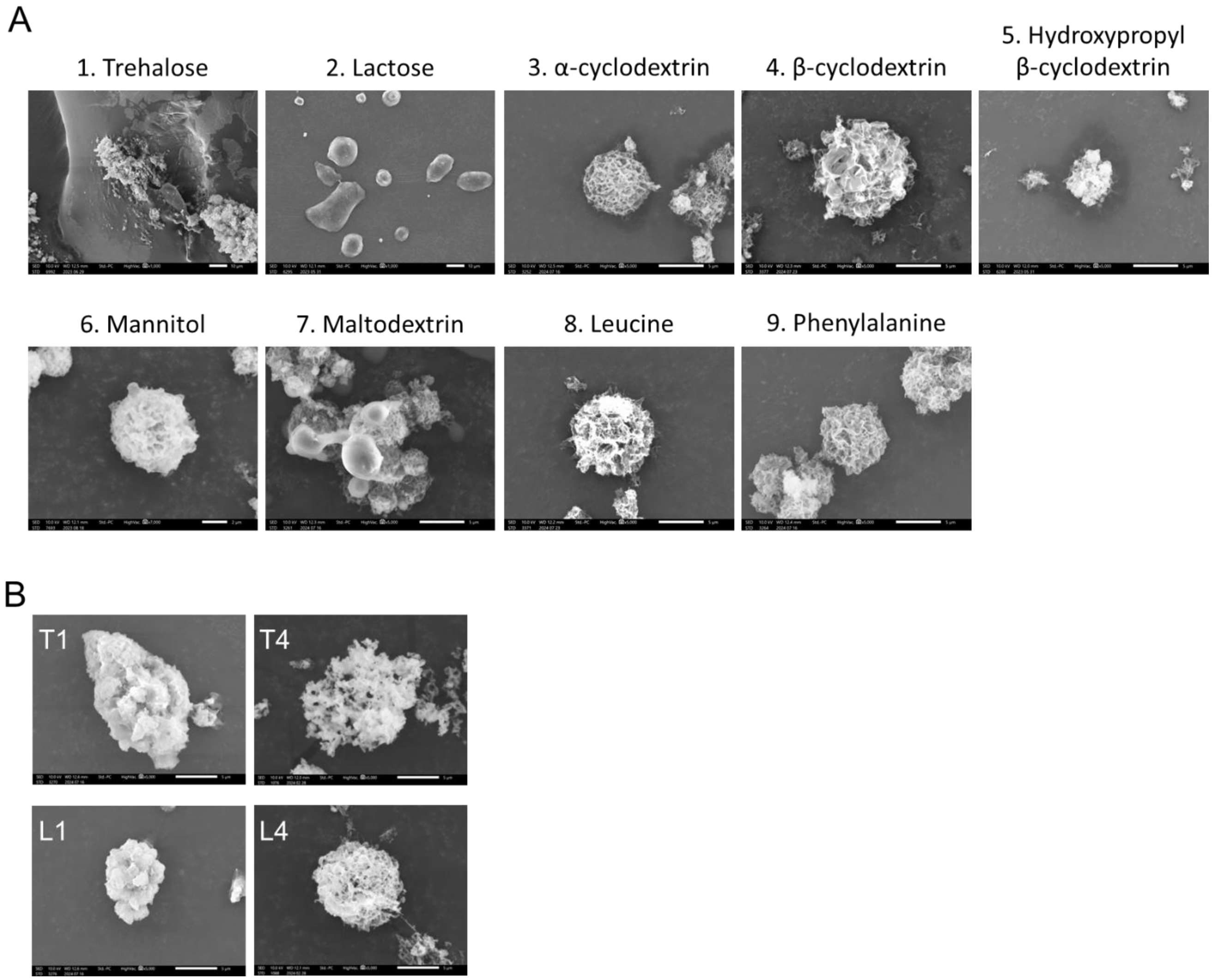

Figure S1. **Representative SEM images of the ACE2 decoy powder via spray freeze drying.** (A) Nine singe excipient formulations listed in Table S1. (B) The mixed formulations, created by blending disaccharides and leucine listed in Table 1. Only T/L1 and T/L4 conditions are shown. Note that trehalose and lactose alone (A) and T/L1 (B) formulations showed wet morphological changes.

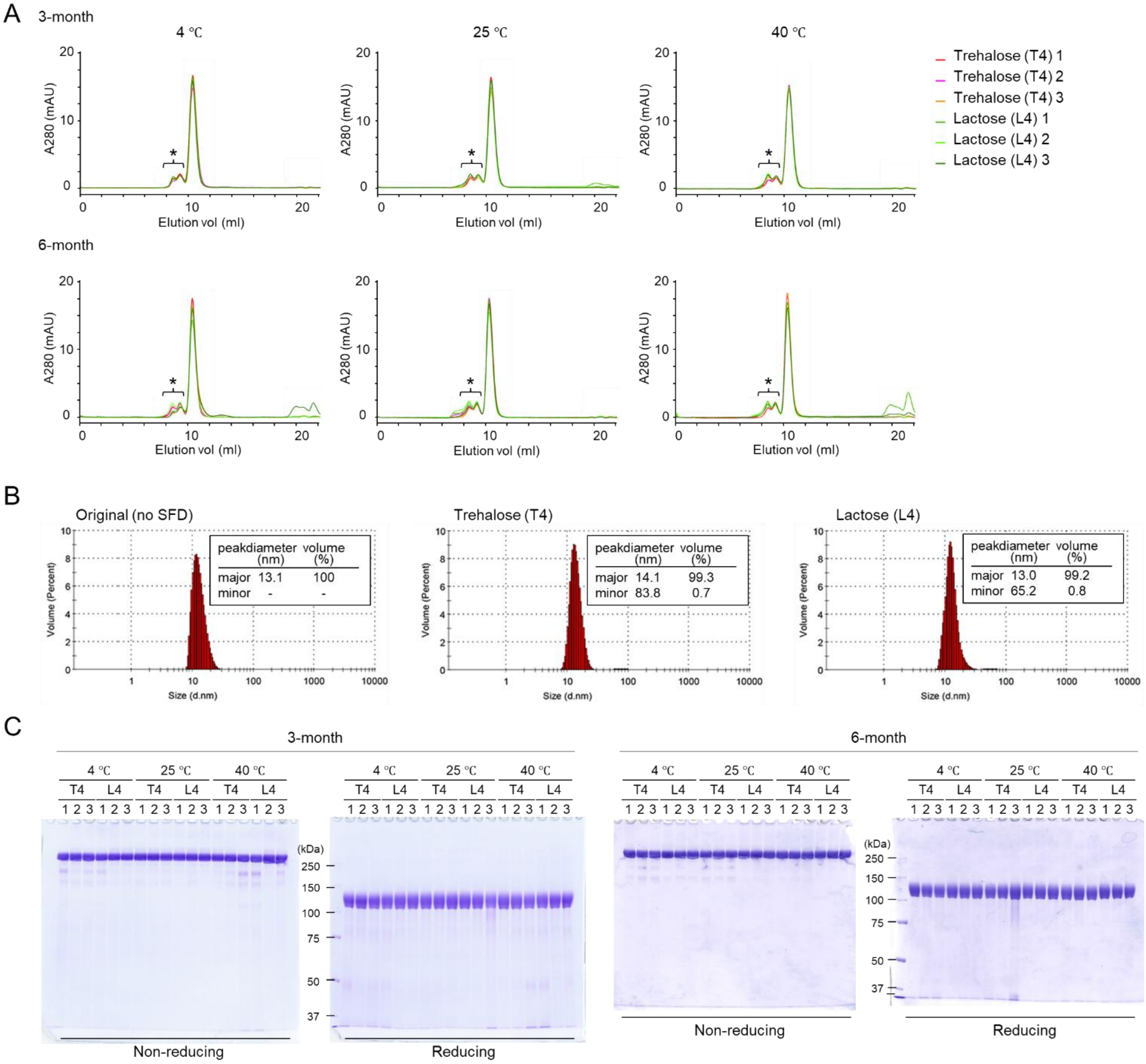

Figure S2. Long-term structural stability of the optimized trehalose or lactose-based dry powder of engineered ACE2 decoy. (A) SEC profiles of reconstituted SFD ACE2 decoy samples after storage under various conditions, related to Figure 3A. For each condition, three replicates were independently prepared and analyzed. Monodispersed decoy protein elutes at 10.3 mL, while aggregated high molecular weight species elute earlier (*). (B) DLS analysis of reconstituted T4 and L4 samples after ~1 year storage at 4°C. Shown are size distribution histograms derived from the average of triplicate measurements. Estimated particle size (nm) and relative abundance (volume %) are shown in the insets. (C) SDS-PAGE analysis of the reconstituted SFD samples to assess the potential SDS-resistant aggregate formation and/or degradation of the ACE2 decoy powder.

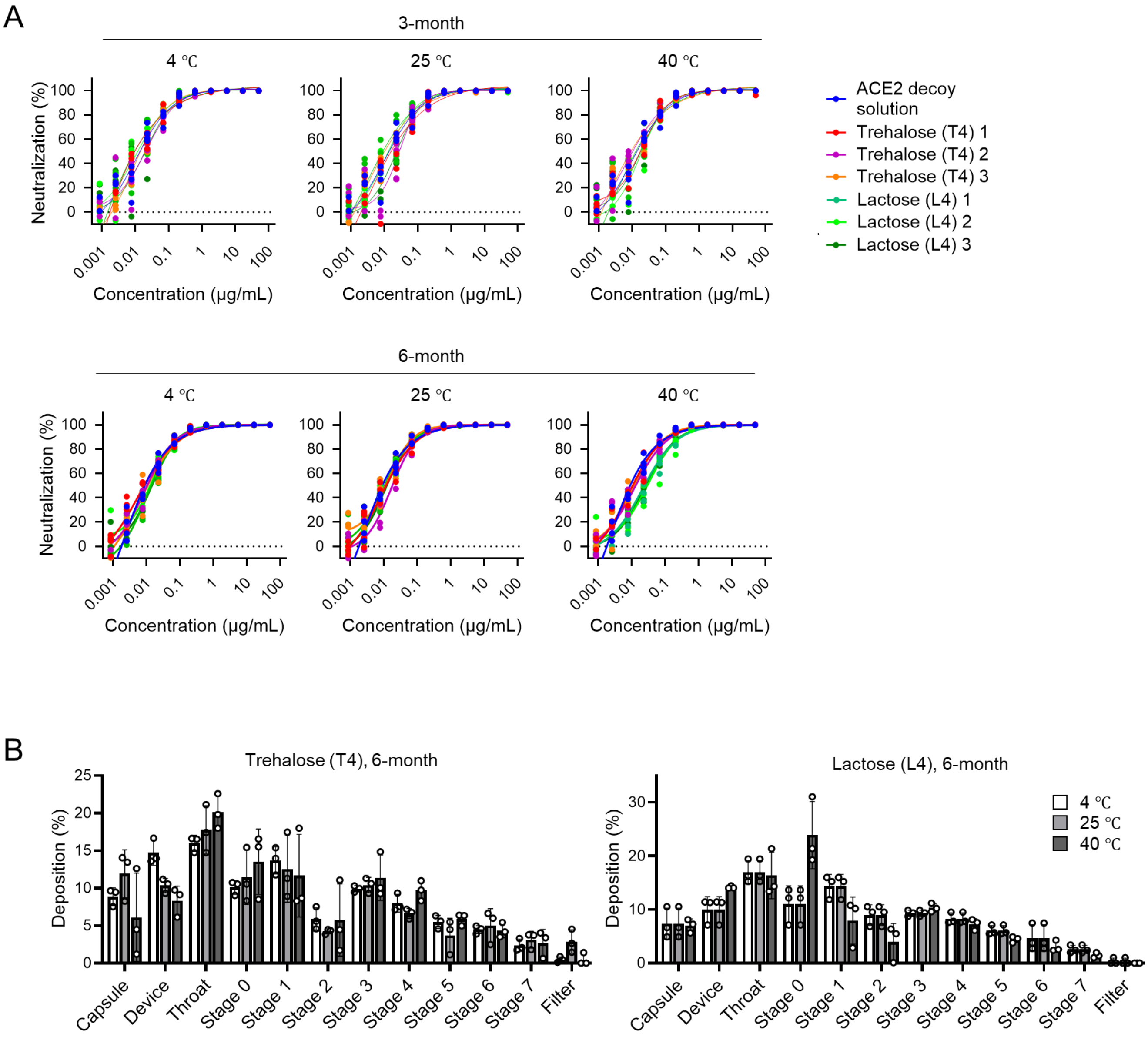

Figure S3. Long-term functional retention of the optimized trehalose or lactose-based dry powder formulation. (A) Neutralizing activity against the ancestral SARS-CoV-2 pseudovirus, related to Figure 3B. (B) Aerodynamic particle distribution of the ACE2 decoy dry powder by a multistage impactor at 6 months, related to Figure 3C. Data are represented as mean ± SEM of n = 3.

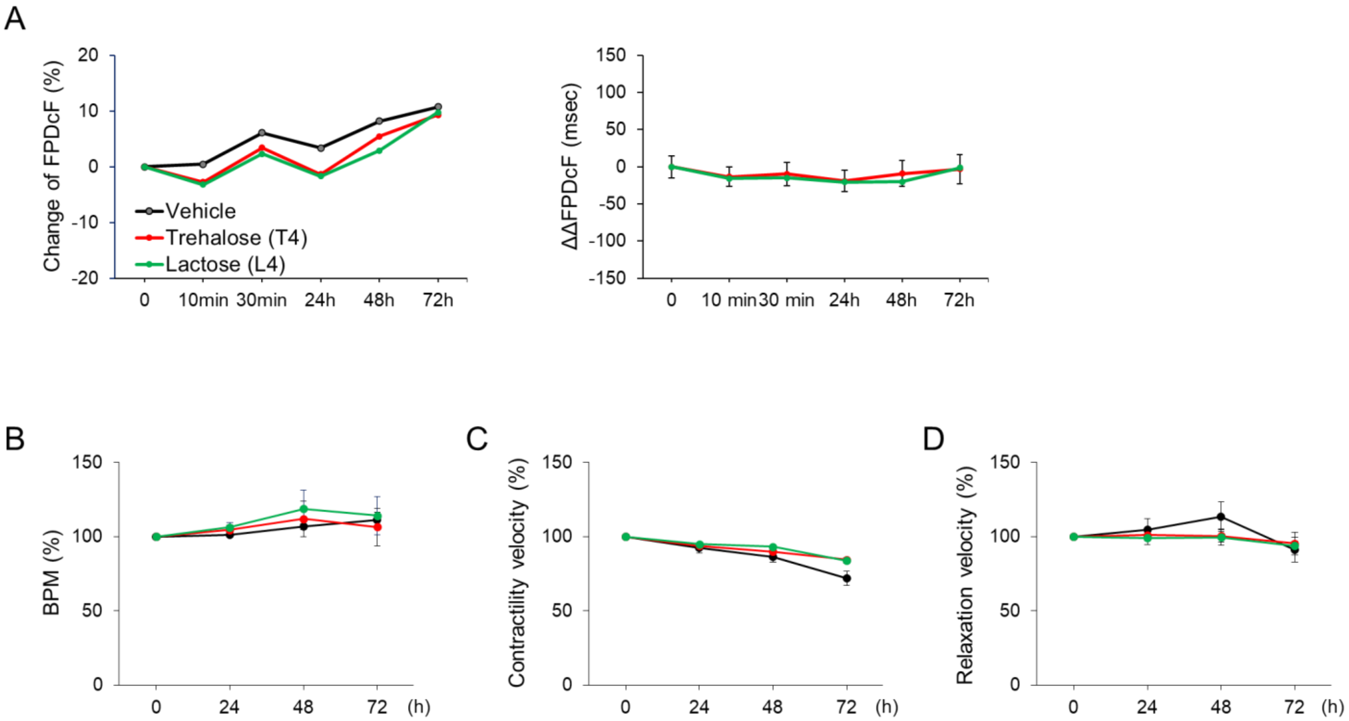

Figure S4. Evaluation of cardiotoxicity of the ACE2 decoy dry powder using human induced pluripotent stem cell (iPS)-derived cardiomyocytes (hiPS-CMs). (A) Change in FPDcF (Field potential duration corrected Fridericia's formula; left) and ΔΔFPDcF (Vehicle-and baseline-corrected FPDcF; right) following treatment with ACE2 dry powders (1 mg/mL) were measured by Field potential recordings. (B to D) Effect of ACE2 dry powders (1 mg/mL) on the contraction parameters were measured by motion analysis (B, beat per minute [BPM]; C, contraction velocity; D, relaxation velocity). Data are represented as mean ± SEM of n = 3.

Table S1. Single excipient formulations of the engineered ACE2 decoy for use in spray freeze drying. Nine excipients (No. 1 to 9) were tested for the spray freeze drying (SFD) of ACE2 decoy. Trehalose and lactose (No. 10 and 11) were also evaluated in the standard freeze drying (FD) condition since these conditions gave highly moisturized particles after SFD. ACE2 decoy solution without any drying procedure (No. 12) was included as a control. All powders were reconstituted in water as described in the Method section and the resultant ACE2 solution were evaluated for their function (neutralizing activity against the ancestral SARS-CoV-2 pseudovirus, expressed as the IC_{50} value) as well as aggregation state (proportion of total high-molecular weight species calculated by peak-integration of the SEC chromatograms as described in the Method). NA, not available.

No	drying condition	Excipient	Ratio (%w/w)		Note	IC_{50} (μg/mL)	Aggregate (%)
			ACE2	Excipient			
1	SFD	Trehalose	5	95	Moisturized	NA	NA
2	SFD	Lactose	5	95	Moisturized	NA	NA
3	SFD	α-cyclodextrin	5	95	Insoluble	NA	NA
4	SFD	β-cyclodextrin	5	95	Insoluble	NA	NA
5	SFD	Hydroxypropyl β-cyclodextrin	5	95		0.0228	29.70
6	SFD	Mannitol	5	95		0.0508	29.63
7	SFD	Maltodextrin	5	95		0.0160	34.80
8	SFD	Leucine	5	95	Insoluble	NA	NA
9	SFD	Phenylalanine	5	95		0.2258	20.34
10	FD	Trehalose	5	95		0.0210	3.70
11	FD	Lactose	5	95		0.0310	4.50
12	none	ACE2 solution	100	0		0.0260	4.20
